# Case report: A case study on the treatment using icaritin soft capsules in combination with lenvatinib achieving impressive PR and stage reduction in unresectable locally progressive pancreatic cancer and a literature review

**DOI:** 10.3389/fgene.2023.1167470

**Published:** 2023-04-20

**Authors:** Xiaolong Liu, Feimin Yang, Dunmao Jia, Xinyu Dong, Yizhuo Zhang, Zhengrong Wu

**Affiliations:** ^1^ Department of General Surgery, Sir Run Run Shaw Hospital, Zhejiang University School of Medicine, Hangzhou, China; ^2^ Department of Nursing, Sir Run Run Shaw Hospital, School of Medicine, Zhejiang University, Hangzhou, China; ^3^ Department of General Surgery, Affiliated Run Run Shaw Hospital, Jiangshan Branch, Harbin Medical University, Quzhou, China

**Keywords:** icaritin soft capsules, lenvatinib, advanced pancreatic cancer, combined treatment, tumor downstaging

## Abstract

**Background:** Pancreatic cancer is one of the most deadly malignancies in the world. It is characterized by rapid progression and a very poor prognosis. The five-year survival rate of pancreatic cancer in China is only 7.2%, which is the lowest among all cancers and the use of combined paclitaxel albumin, capecitabine, and digital has been the clinical standard treatment for advanced pancreatic cancer since 1997. Also, the application of multidrug combinations is often limited by the toxicity of chemotherapy. Therefore, there is an urgent need for a more appropriate and less toxic treatment modality for pancreatic cancer.

**Case presentation:** The patient was a 79-year-old woman, admitted to the hospital with a diagnosis of unresectable locally advanced pancreatic cancer (T3N0M0, stage IIA), with its imaging showing overgrowth of SMV involvement and unresectable reconstruction of the posterior vein after evaluation. As the patient refused chemotherapy, lenvatinib (8 mg/time, qd) and icaritin soft capsules (three tablets/time, bid) were recommended according to our past experience and a few clinical research cases. The tumor lesion was greatly reduced by 57.5% after the treatment, and the extent of vascular involvement also decreased. The aforementioned medication resulted in a significant downstaging of the patient’s tumor.

**Conclusion:** Better results were achieved in the treatment with icaritin soft capsules and lenvatinib in this case. Because of its less toxic effect on the liver and kidney and bone marrow suppression, it was suitable to combine icaritin soft capsules with targeted drugs for treating intermediate and advanced malignancies, which brings hope to patients who cannot or refuse to take chemotherapy.

## Preface

Today, pancreatic cancer is one of the deadliest malignancies. The global tumor registry data of 2020 show that pancreatic cancer ranks 12th in incidence but 7th in mortality among malignancies (Hu et al., 2021). It is mostly characterized by rapid progression and a very poor prognosis. The five-year survival rate of pancreatic cancer in China is only 7.2%, the lowest among all tumor types (Zhao et al., 2019). Most patients are diagnosed with locally advanced pancreatic cancer or distant metastases when the tumor is detected, so only 15%–20% of patients have the opportunity to accept surgical treatment with a better prognosis (Van Veldhuisen et al., 2019). A total of 95,000 new cases of pancreatic cancer were diagnosed in China in 2015, while 85,000 deaths occurred, with generally higher morbidity and mortality rates in men and in urban areas (Jia et al., 2018).

Gemcitabine has been the standard chemotherapy for advanced pancreatic cancer since 1997. Several phase III clinical trials have tried chemotherapy with gemcitabine to improve outcomes. However, most of these trials failed to show an improvement in overall survival, except for two studies. A phase III trial of erlotinib and gemcitabine showed very limited improvement compared to gemcitabine alone (Moore et al., 2007). Also, in the ACCORD 11 study, better results were observed with FOLFIRINOX chemotherapy (five-fluorouracil, oxaliplatin, and irinotecan with folinic acid), which showed an improvement in overall survival by more than four months (from 6.8 to 11.1 months) compared to gemcitabine (Conroy et al., 2010). However, the significant toxicity of multidrug chemotherapy often limits its use to the extent that some patients refuse it. Therefore, there is an urgent need for a more efficient and less toxic treatment modality for pancreatic cancer.

Studies have concluded that most pancreatic cancers are blood-deprived tumors with insignificant angiogenesis, and therefore, there is little data about the anti-angiogenic drugs for clinical treatment. However, one study showed that the ORR of lenvatinib for the treatment of pancreatic neuroendocrine tumors was up to 44.2% and the DCR was 96.2%, which proves its effectiveness (Capdevila et al., 2021). Other cases of target-free combination therapy for pancreatic tumors have also been reported, such as a 55-year-old pancreatic cancer patient (cT4N1M1) with liver and lung metastases who carried ERBB2 mutation and had high tumor mutational load (TMB) being treated with lenvatinib in combination with pembrolizumab; it achieved partial remission for up to 5 months after a series of treatments failed (Chen et al., 2019). A 48-year-old patient with metastatic pancreatic alveolar cell carcinoma treated with lenvatinib and sintilimab demonstrated significant tumor remission and long-term progression-free survival (>21 months) (Qin et al., 2021). The data previously discussed suggest that lenvatinib and immunologic agents may be effective in the clinical treatment of pancreatic cancer.

Icaritin soft capsules are an original small-molecule immunomodulator with independent intellectual property rights in China; it is a first-in-class original drug in the world, and it was approved by China’s National Medical Products Administration (NMPA) in January 2022. The results of its preclinical studies and *in vivo* pharmacodynamic study showed that epimedium had a significant dose-effect positive correlation in inhibiting tumor growth in the human-derived hepatocellular carcinoma Hep G2 mouse liver, which is an *in situ* transplantation tumor model, and also had different inhibition of tumor growth in the human-derived breast cancer BCAP-37, human-derived prostate cancer PC-3, and other subcutaneous transplantation tumor models, which also showed a certain quantitative-effect relationship (Huang et al., 2007; Tong et al., 2011; Sun et al., 2021), confirming its significant and broad-spectrum antitumor activity. As a novel small-molecule immunomodulator, it can inhibit inflammatory signaling pathways, reduce the release of inflammatory factors, enhance antigen presentation, decrease expression of PD-L1 and MDSC, increase T-cell termination, and improve the immune microenvironment of tumors through dual activation of intrinsic and adaptive immunity. The current indication of the icaritin soft capsule is approved for the first-line treatment of hepatocellular carcinoma (Guo et al., 2011). As far as we know, there are many studies about liver cancer treatment with icaritin soft capsule, and no related research concerning different stages of pancreatic cancer treatment with icaritin soft capsule in practice.

This article reports a case of a patient with locally progressive unresectable pancreatic cancer who refused chemotherapy and achieved PR after treatment with lenvatinib and icaritin soft capsules with satisfactory efficacy and controlled safety.

## Case presentation

The patient is a 79-year-old woman admitted to our hospital in mid-August 2022 for “distension and pain in the upper and middle abdomen for 1 week and pancreatic occupancy for 6 days.” In fact, the patient could maintain a normal life and take care of herself except for distension and pain in the upper and middle abdomen without any cause, accompanied by nausea and a small amount of vomiting, which were mostly gastric contents. The KPS score of the patient was approximately 80. The aforementioned condition could evidently be aggravated by hunger and was relieved by eating small amounts of food. There was no fever, chills, diarrhea, or black stool. At first, she went to the local hospital to find elevated CA 19-9 levels (details unknown), and abdominal ultrasonography suggested an occupancy in the head of the pancreas. For further diagnosis and treatment, the patient came to our hospital with a diagnosis of pancreatic mass. The patient denied a past history of hypertension, diabetes mellitus, hepatitis, tuberculosis, etc. and denied any history of surgical trauma. The hospital conducted a physical examination as follows: T 36.9°C, R 19°bpm, HR 85°bpm, BP 159/72 mmHg, and pain score 1. The patient was conscious and mentally competent. The skin and sclera were not yellowish and without enlarged superficial lymph nodes, with clear breath sounds in both lungs and uniform heart rhythm without cardiac murmur. The abdomen was flat with normal bowel sounds and negative shifting dullness. The whole abdomen was soft without abnormal masses and pressure pain or rebound pain. There was no edema in both lower extremities, and the pathological features were negative.

The supplementary examination included the blood count: WBC, 6.0*10^9/L; Neo, 3.32*10^9/L; HB, 128 g/L; and Plt, 254*10^9/L.

**FIGURE 1:**
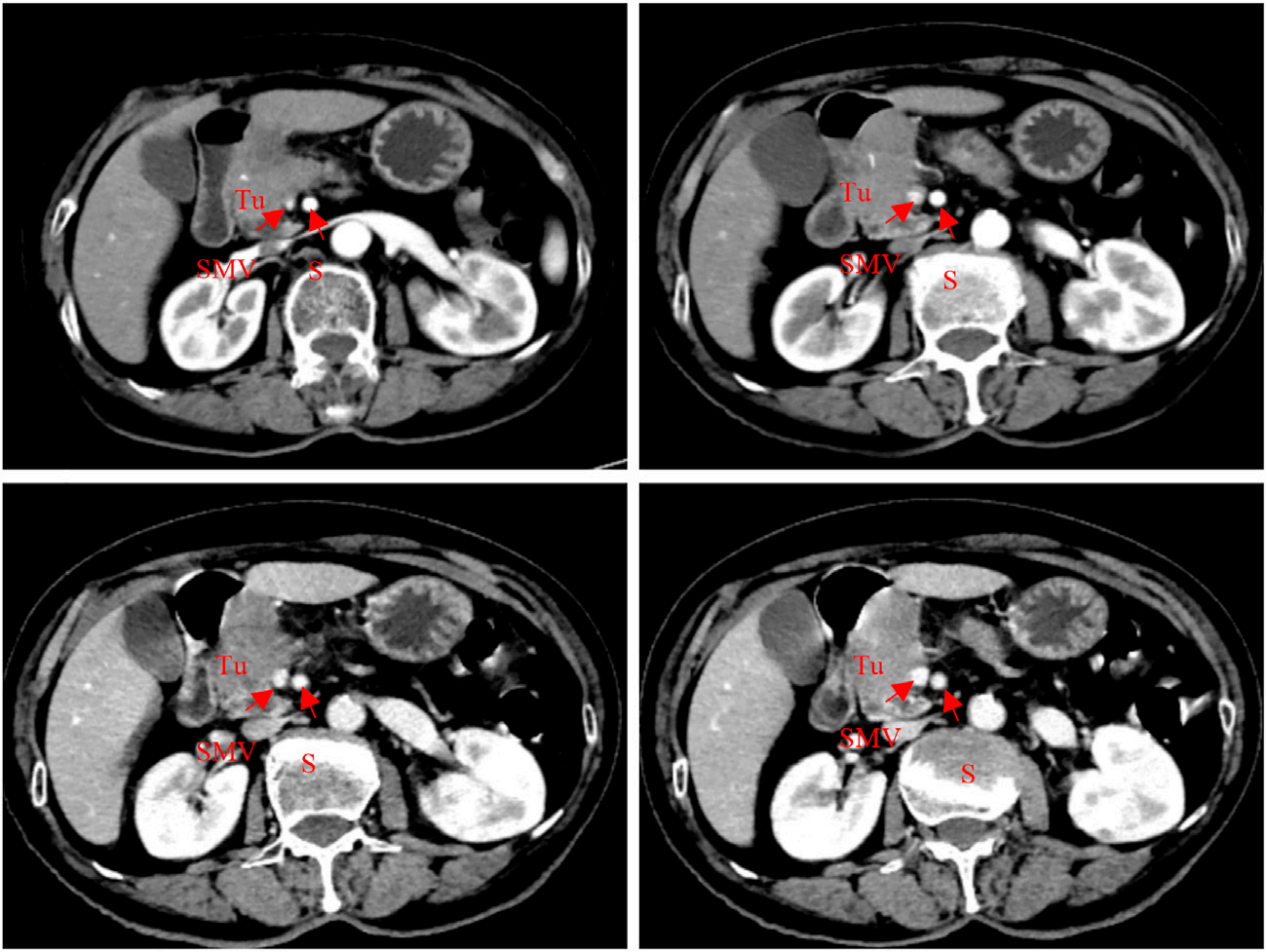
Abdominal CT scan (2022-08-05): a soft tissue mass shadow was seen in the pancreas head, approximately 57*32 mm; meanwhile, the gastroduodenal artery and superior mesenteric vein were also involved.

Blood biochemistry was reported as follows: K, 3.78 mmol/L; ALT, 19 U/L; AST, 17 U/L; AKP, 123 U/L; γGGT, 45 U/L; ALB, 42.9 g/L; TBIL, 6.3 μmol/L; and DBIL, 1.0 μmol/L. Blood glucose was 11.4 mmol/L. Tumor markers included CA 19-9, 69.8 IU/mL; CEA, 1.39 ng/mL; CA 15-3, 10.60 U/mL; CA 125, 14.30 U/mL; and AFP, 1.77 ng/mL. CT scan images of admission (Figure 1) showed occupancy in the pancreas head, which was considered to be cancer, with an involvement of the gastroduodenal artery and the superior mesenteric vein, and a soft tissue mass shadow was observed in the pancreas head, approximately 57*32 mm.

Ultrasound endoscopy showed a well-defined and irregular hypoechoic mass in the pancreatic head with a vascular invasion of approximately 43*29 mm. The preliminary diagnosis was considered pancreatic malignancy (cT3N0M0, stage IIA), which indicated the clinical diagnosis stage. Due to excessive SMV involvement and evaluation of posterior venous unresectable reconstruction, the disease was diagnosed as locally progressive unresectable pancreatic cancer. The current standard first-line treatment for advanced unresectable pancreatic cancer is based on a gemcitabine regimen. However, the patient insistently refused chemotherapy after repeated communication and explanation, and based on its immune regulation mechanism, combining our previous medication experience with the icaritin soft capsule, we tried to recommend lenvatinib and icaritin soft capsule for treatment. To the best of our knowledge, no case study was found to be reported to treat pancreatic cancer until now.

**FIGURE 2:**
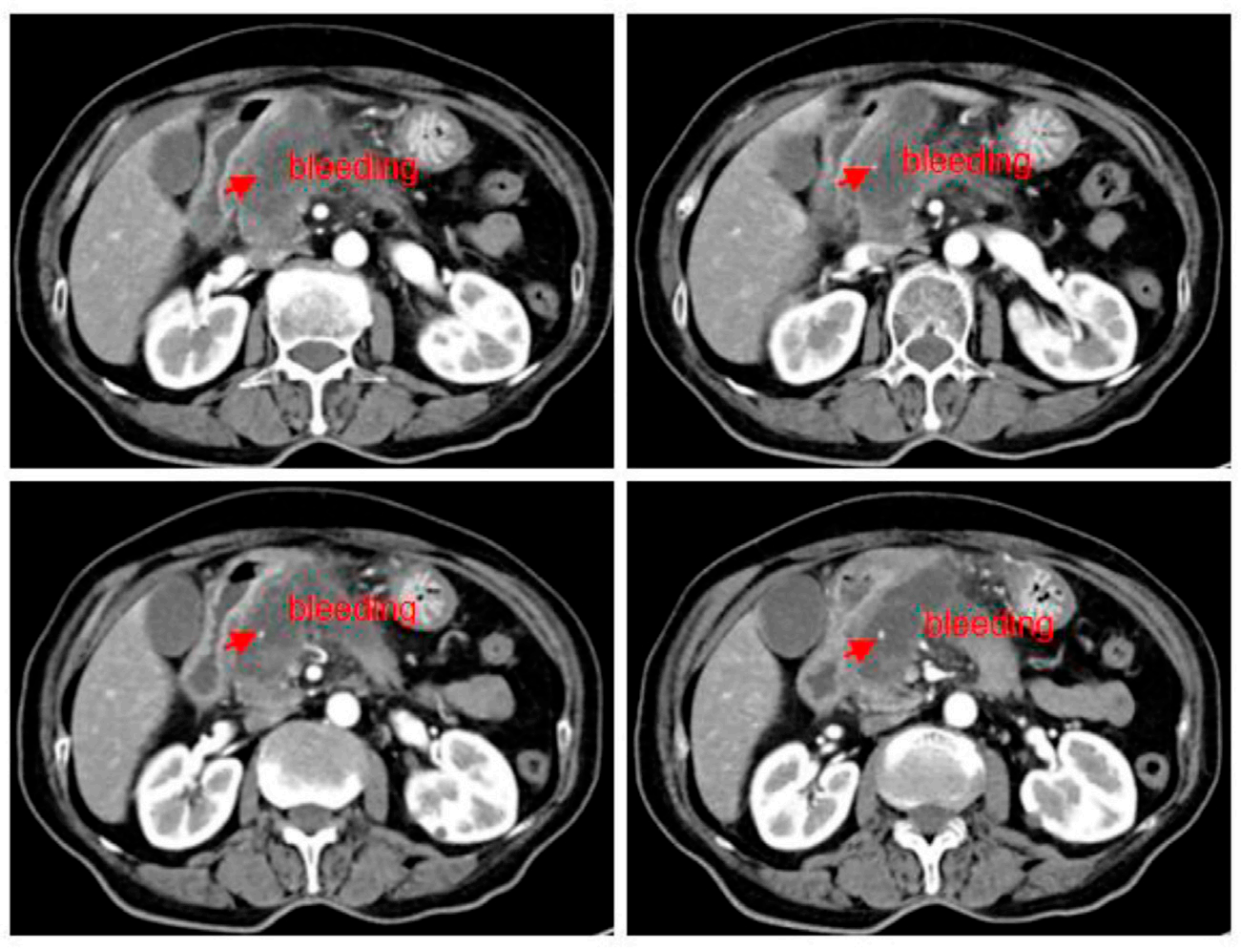
Abdominal CT scan (2022.08.27): a mass of shadow in the neck of the pancreatic head, approximately 57*32 mm; tumor with cystic necrosis was considered as a small amount of local bleeding.

The patient underwent a CT review after 1 month of medication, and although the huge mass did not shrink and significant cystic necrosis was visible inside, in the meantime, the significant spillage of the contrast agent could be observed, suggesting the possibility of bleeding inside a tumor. It was inferred that the disease has been controlled (Figure 2). Since the tumor envelope was still intact, it was considered that the risk of progressive bleeding could be avoided by self-compression to stop the bleeding. Generally speaking, short-term and small amounts of bleeding have limited harm, but it could lead to death with a large amount of bleeding. The patient was discharged after conservative treatment in the hospital.

**FIGURE 3:**
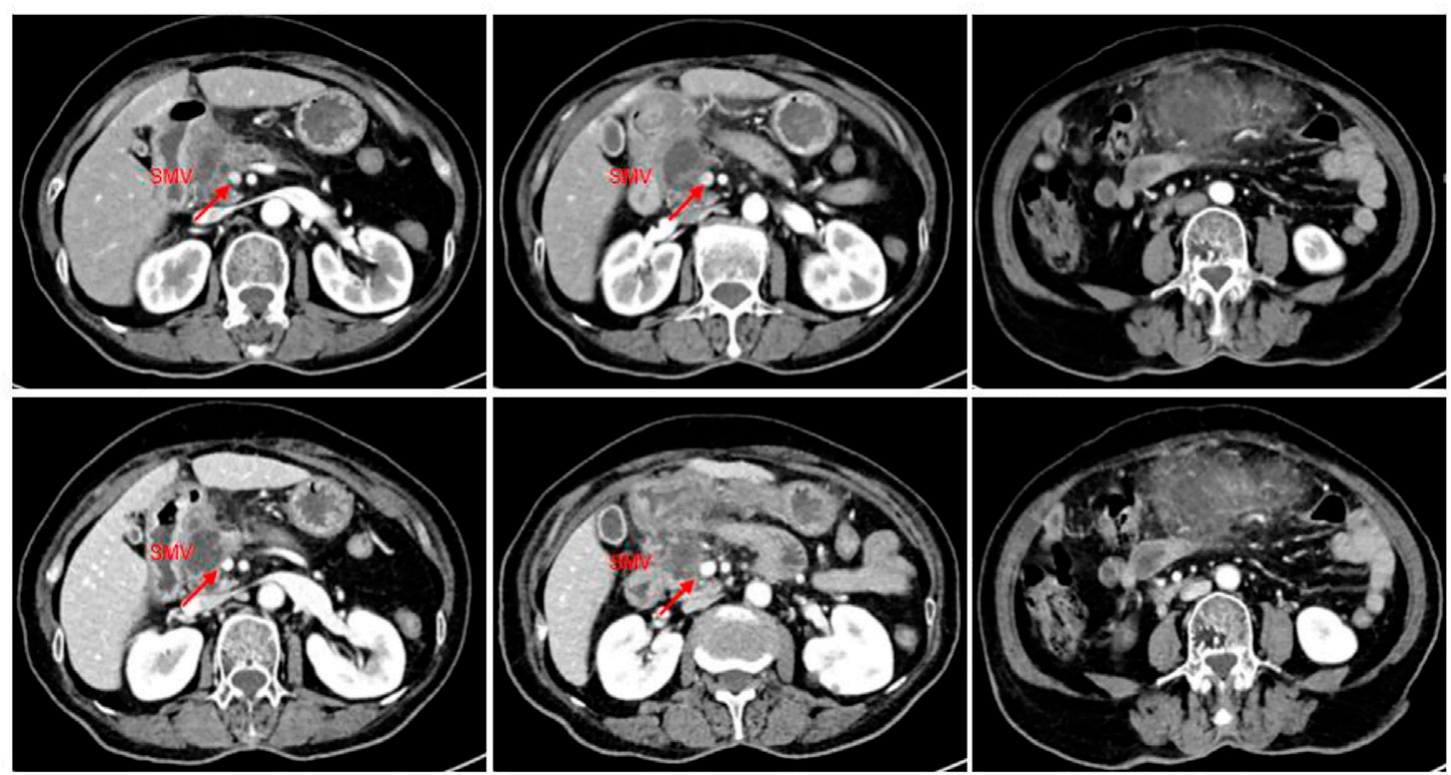
CT imaging of the abdomen (2022.9.26): a slightly hypodense mass of approximately 35*28 mm with mild enhancement at the margin was observed in the neck of the pancreatic head, and the right wall of the proximal end of the adjacent superior mesenteric vein was involved with a slightly narrower lumen. The tumor was 52% smaller than before treatment.

The patient came to our hospital for the second time due to sudden abdominal pain. The routine blood tests showed WBC, 12.0*10^9^/L; Neo, 9.82*10^9^/L; CRP, 188.5 mg/L; blood amylase, 239 IU/L; and lipase, 231.10 IU/L; all of the aforementioned indicators were elevated. The tumor biomarker of CA 19-9 showed a decrease from 70 IU/mL to 30 IU/mL, and the imaging showed a 52% reduction in tumor size (35*28 mm), a reduction in superior mesenteric vein (SMV) involvement, and significant relief of vascular stenosis. The efficacy of lenvatinib and icaritin soft capsules was evaluated comprehensively and reached PR with significant tumor remission after 2 months (Figure 3). Anti-infective and amylase-lowering symptomatic treatment was given for acute pancreatitis after her admission into the hospital.

**FIGURE 4:**
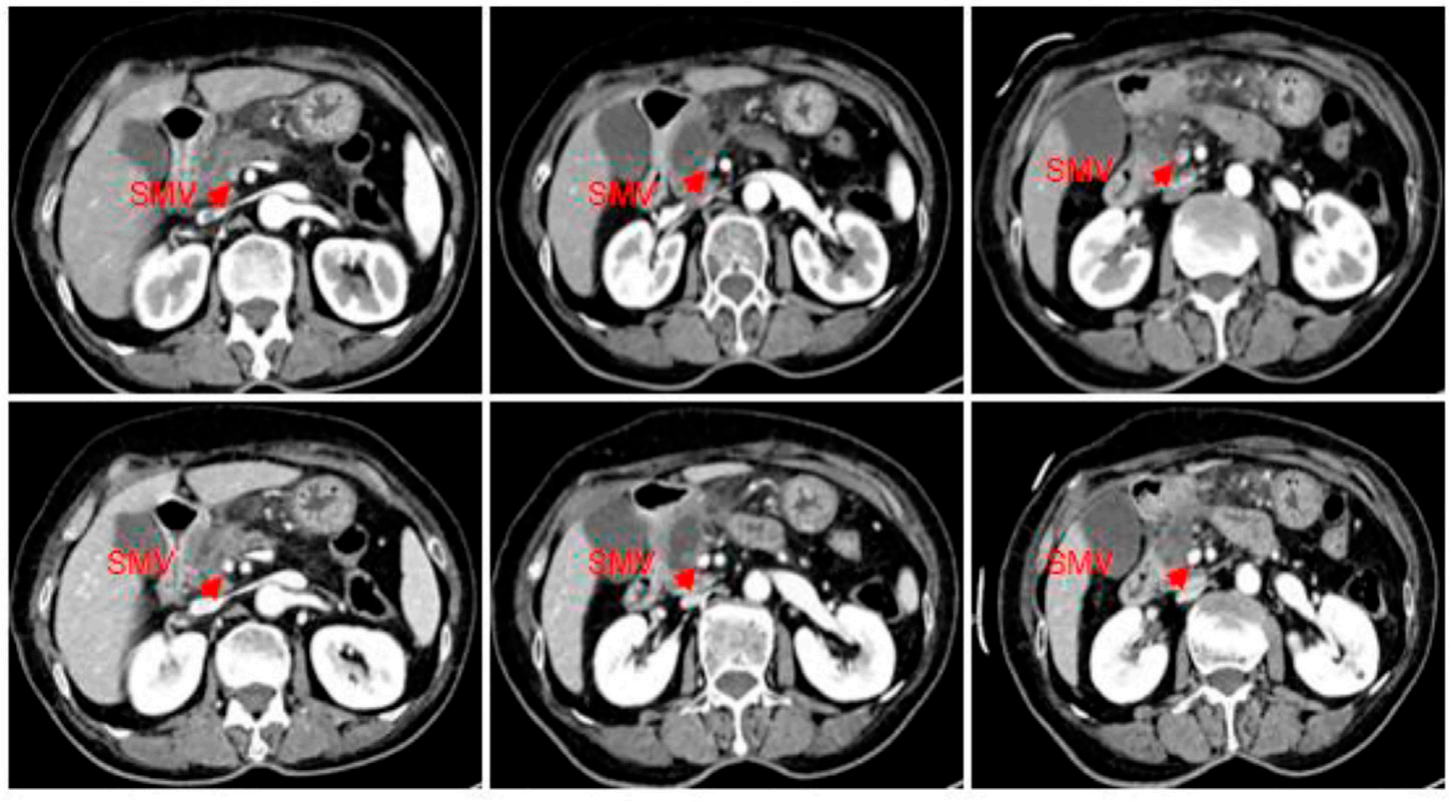
CT scan of the abdomen (2022.10.15): a slightly hypodense mass of approximately 35*28 mm with mild edge enhancement was seen in the neck of the pancreatic head, with a slightly reduced lesion compared to the last imaging (2022.09.26), and with a relief of the proximal stenosis in the adjacent SMV. It showed better improvement than before for the vague edema and thickening of the lateral wall of the greater curvature of the gastric antrum and the colorectal wall, and the vague inflammatory exudation of the gastrocolic ligament. The tumor was 20% smaller than in the last review.

On 15 October 2022, the patient was readmitted to the hospital again with sudden onset of abdominal pain for 1 day, and routine blood showed WBC, 14.7*10^9^/L; Neo, 11.98*10^9^/L; CRP, 156.3 mg/L; blood amylase, 211.6 IU/L; and lipase, 224.0 IU/L. CT of the abdomen showed a diffuse inflammatory exudate below the pancreas, suggesting secondary acute pancreatitis (Figure 4). Also, the tumor lesion was approximately 20% smaller than last time and 57.5% smaller overall than before treatment. The vascular involvement was also reduced and the proximal stenosis of SMV was also relieved with a better contour. The patient’s condition was regarded to be locally advanced pancreatic cancer as before, and the surgical assessment met the criteria for SMV resection and reconstruction (Figure 4). Therefore, its TNM stage was downgraded from T3N0M0 (stage IIA) before the treatment to T2N0M0 (stage IB) after the treatment, and surgery was recommended as an effective treatment choice. Meanwhile, the patient requested the surgery after another two cycles of medication due to the results being beyond her expectation. The patient was admitted to the hospital only for an anti-infective symptomatic treatment of secondary acute pancreatitis.

**FIGURE 5:**
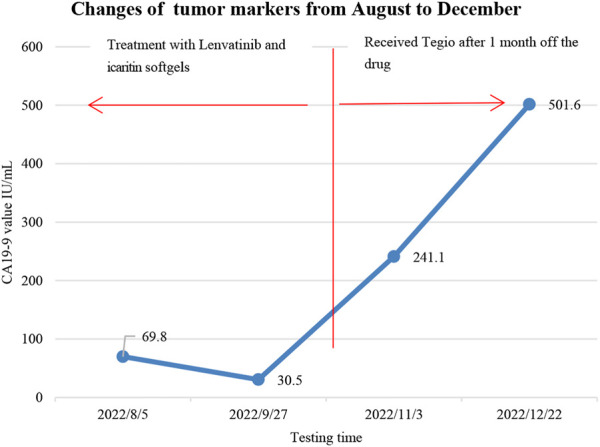
Changes in tumor markers: the patient took two drugs for 2 months between August and stopped them in early October for financial reasons, later replaced them with Tegio in early November, and followed up about 3 months after the drug’s withdrawal.

Unfortunately, the patient stopped taking these two drugs for financial reasons and was re-examined 1 month later, which showed an increase of 1 cm in the active component of the primary pancreatic lesion (original necrotic part), an increment of 2 cm in the tumor located in the pancreatic hook, and multiple metastases in the hepatoduodenal ligament and the hepatogastric ligament based on the results of abdominal CT imaging. Also, increased tumor biomarkers, such as CA 19-9 (241.1 U/mL) and ferritin (686.0 ng/mL), on November 3 suggested tumor progression. Later, the patient took Tegio as treatment, and CA 19-9 increased to 501.6 U/mL in December without a CT review (Figure 5). Also, the patient is still alive today.

## Treatment summary

Icaritin soft capsules are now widely used as an immunomodulatory agent in the treatment of solid tumors. In this case, a 79-year-old woman with nausea and vomiting was found to have elevated CA 19-9 at a local hospital, while an ultrasonic examination showed an occupied lesion in the patient’s pancreatic head. In our hospital, it just presented with the symptoms or signs of slightly elevated blood pressure, blood glucose, and mild abdominal pain. The patient was initially diagnosed with pancreatic cancer with T3N0M0 (stage IIA). The evaluation revealed a tumor invading an SMV, which was unfit for removal and reconstruction, and it can be classified as an unresectable, locally advanced pancreatic cancer. Generally, pancreatic cancer at this stage can be treated with gemcitabine combined with albumin paclitaxel (GN) or gemcitabine and cisplatin (GP) according to the new guidelines recommended by CSCO, whose efficiency can reach 20%–30% (Cui et al., 2022). However, the fact lies in the low validity as expected, and some serious side effects with chemotherapy. Therefore, the patient’s family insisted on refusing chemotherapy. The choice of icaritin soft capsules and lenvatinib was mainly based on the following reasons:

1) Due to economic reasons, the patient refused gene sequencing, including the BRAC1/2 gene and other tumor-associated genes, and no targeted drugs could be chosen and applied without the definite gene status of the BRAC1/2 gene; meanwhile, the patient refused radiotherapy.
2) Icaritin soft capsules are a broad-spectrum immunomodulator for solid tumors, with enough evidence for a favorable experience of clinical application and good security.
3) There are some data to support the use of lenvatinib in the clinical treatment of pancreatic cancer.

Pancreatic cancer is generally a blood-deprived tumor with insignificant angiogenesis; therefore, the role of anti-angiogenic drugs in the treatment of pancreatic cancer remains to be proven. Studies have shown that all types of tumors require vascularization to provide the oxygen and nutrients, which tumors need to grow (Craven et al., 2016). According to Haltly’s research, the vasculature was generally divided into visualized tumor vessels and microscopic microvasculature. Now, the microvessel density is thought to be a better indicator of tumor metabolic load than visual angiogenesis. Therefore, it is believed that tumors lacking blood supply can also benefit from anti-angiogenic therapy (Lynn et al., 2002). Although there are fewer visualized vessels in pancreatic cancer, a higher microvessel density suggests the possible benefits of the anti-VEGF treatment. However, the clinical practice has shown that small-molecule TKIs, including bevacizumab and aparatinib in combination with chemotherapy for pancreatic cancer, did not meet the expectations due to insignificant OS prolongation and even shorter survival than chemotherapy alone in some cases (Kindler et al., 2010; Gonçalves et al., 2012; Bergmann et al., 2015; Kim et al., 2015). This may be related to the fact that targeted drugs increase the side effects and prevent patients from the full course of chemotherapy, which leads to poor treatment effects.

Generally, due to the complex mechanism of tumorigenesis and metastasis, combination therapy can inhibit tumors from multiple mechanisms to achieve better clinical results; therefore, multiple combined medication schemes are recommended by NCCN, ASCO, and CSCO guidelines in the clinical practice at present. There are data about the treatment of pancreatic cancer with lenvatinib. In fact, lenvatinib is a synthetic multi-target inhibitor of tyrosine kinase with oral activity, which is most effective for the VEGFR2 (KDR)/VEGFR3(Flt-4) gene mutation of cancer. Also, we think that it is an anti-tumor drug, not an immune modulator. There is a report that the ORR reached 44.2% with lenvatinib after the treatment of the pancreatic neuroendocrine tumor, and DCR was up to 96.2%, which showed higher efficiency but a poor safety profile. The common adverse events often included fatigue, hypertension, and diarrhea, and approximately 93.7% of patients would have dose reduction requirements or treatment interruption (Capdevila et al., 2021). Another study also exhibited its inhibition focused on the growth of pancreatic cancer graft tumors and also mentioned that microvascular density may be related to the efficacy of anti-angiogenic drugs (Yamamoto et al., 2014). This inferred the therapeutic effects of pancreatic cancer with lenvatinib.

There are few clinical precedents of targeted-drug monotherapy for pancreatic cancer until now. In this case, based on two case reports of lenvatinib and immune-related drugs of pancreatic tumors and our experience (Yi et al., 2019), and the patient’s opinion of refusing chemotherapy, icaritin soft capsule, as a new small-molecule immunomodulator, and lenvatinib was used in the treatment for the patient. It is believed that immunomodulation of icaritin soft capsule plays an important role in tumor cells and the microenvironment, including 1) icaritin suppresses IL-6/JAK2/STAT3 signaling pathway by inhibiting JAK2 and STAT3 phosphorylation, leading to downregulation of its downstream-related genes (Zhu et al., 2015); 2) directly binds MyD88/IKKα and inhibits the TLR-MyD88-IKK-NFκB-signaling pathway, which in turn reduces TNF-α, IL-6, and other factors’ production and downregulates the IL-6/JAK2/STAT3-signaling pathway (Li et al., 2021). It is shown that the two signaling pathways aforementioned are complementary and can exhibit anti-tumor effects by downregulating inflammatory factors, such as TNF-α, IL-6, and PD-L1 expression. The results demonstrated that the number and proportion of CD8^+^ T cells in tumor tissues of mice were significantly increased, and the proportion of MDSCs was significantly reduced in the icaritin soft capsule group in the related immune microenvironment studies, indicating that it not only increased the number and activity of CD8^+^ T cells but also effectively reduced the proportion of immunosuppressive cell MDSCs (Hao et al., 2019). It is concluded that restoring the ability of CD8^+^ T cells could contribute to producing IFN-γ and improving the tumor microenvironment to prevent tumor growth.

The patient was re-examined after 1 month of medication, and CT showed that the lesion did not shrink but a larger internal cystic necrosis and notable contrast spillage were seen, suggesting the presence of tumor bleeding and the possibility of tumor remission. After the whole treatment, significant tumor remission and a good quality of life improvement were observed in this patient, and it proved the treatment effective. This may be related to the combined potency of the two drugs. Cytokine IL-6 has various pro-tumor activities, such as promoting the release of angiogenic factors leading to neovascularization (Fisher et al., 2014); therefore, downregulation of IL-6 expression may enhance the inhibition of tumor microangiogenesis and thus increase its effects of anti-angiogenic drugs.

The commonly mutated genes in pancreatic cancer include BRCA1/2, CDKN2A, PALB2, ATM, TP53, STK11, and PRSS1. Currently, patients with a family history of pancreatic cancer are mostly recommended to undergo BRCA1/2 and other related genetic tests to clarify the possibility of tumor heritability, and to help targeted-drug screening and clinical treatment on the other hand. Lenvatinib is a receptor tyrosine kinase (RTK) inhibitor that inhibits the VEGF receptor VEGFR1/2/3 kinase activity and also inhibits pathological tumor angiogenesis, thereby inhibiting tumor growth and progression. It is approved for unresectable hepatocellular carcinoma primarily based on the results of the non-inferiority, multicenter randomized REFLECT Phase III clinical study compared with sorafenib. The Phase II clinical trial of GETNE1509 with lenvatinib also demonstrated efficacy against progressive advanced pancreatic and gastrointestinal neuroendocrine cancers (Capdevila et al., 2021). There are details about gene mutations in pancreatic cancer (Table 1). Another phase II clinical study of lenvatinib in combination with a PD1 inhibitor for unresectable cholangiocarcinoma has also shown good efficacy and promise (Zhang et al., 2021). Because of the indications for advanced hepatocellular carcinoma of lenvatinib, it is currently less used in the clinical treatment of pancreatic cancer. Therefore, there is no expert consensus or guidelines for selecting lenvatinib for locally progressive unresectable pancreatic cancer based on its tumor-related driver gene by gene sequencing. Nevertheless, high-throughput sequencing will undoubtedly become a key cornerstone of pharmacogenomics and individualized therapy during the treatment of tumors (Morganti et al., 2019). In this case, the patient had significant efficacy with two cycles of lenvatinib and icaritin soft capsules, considering the possible existence of VEGFR1/2/3 gene variants and their benefits. In the future, the treatment of pancreatic cancer may require more high-throughput sequencing as an important means and tool for treatment selection. With the discovery of more tumor-driver genes and the research and application of related targeted drugs, the treatment of pancreatic cancer and its clinical prognosis can be expected. The tumor is actually a chronic inflammatory process accompanied by changes in various inflammatory factors. Because we paid more attention to clinical symptoms during the treatment course, the related clinical symptoms, imaging findings, and hematological examination could only indicate therapeutic effects, while neglecting the tumor microenvironment change without conducting for the inflammatory factor test. In the future, more observation and research should be conducted on the changes in relevant inflammatory factors in the tumor treatment.

**TABLE 1:** Common pancreatic cancer-related gene variations and possible effective targeted drugs (some table contents based on the public data of COSMIC).

Common pancreatic cancer-related gene	Point mutation		Copy number variation		Gene expression		Targeted drug
	% mutated	Tested	Variant %	Tested	% regulated	Tested	
BRCA1	2.32	2,891	0.22	898	6.15 (Over expressed)	179	PARP inhibitors: oplaparib, rucaparib, and niraparib
					0.56 (Under expressed)		
BRCA2	3.83	3,079			5.59 (Over expressed)	179	The same as aforementioned
CDKN2A	7.64	4,582	4.68	898	12.29 (Over expressed)	179	CDk4/6 inhibitors: palbociclib
PALB2	1.27	2,828			4.47 (Over expressed)	179	May benefit from PARP inhibitors treatment
					1.68 (Under expressed)		
ATM	5.7	3,649			3.35 (Over expressed)	179	May benefit from oplaparib treatment
TP53	38.53	5,076			7.82 (Over expressed)	179	
					9.5 (Under expressed)		
STK11	1.81	3,932	0.22	898	3.91 (Over expressed)	179	PARP inhibitors: bemcentinib + pembrolizumab
					10.61 (Under expressed)		
PRSS1	0.65	2,011			3.35 (Over expressed)	179	

At present, tumor immunotherapy is widely carried out; unfortunately, the effect is not clear with many clinical studies of immunotherapy in combination with chemotherapy, chemoradiotherapy, vaccines, and cytokine antagonism in patients with pancreatic cancer (Sahin et al., 2017). Generally, the microenvironment of pancreatic cancer is thought to create an immunosuppressive environment for its low immunogenicity, and there is currently no immunotherapy approved for these patients with pancreatic cancer. Also, the FDA approved the use of pembrolizumab in the treatment of microsatellite unstable cancers unrelated to the type of cancers, which seems to depend on the synergistic effect with increased response rates when a combinatorial approach of immunotherapy in conjunction with other modalities is being used (Schizas et al., 2020; Zhao and Liu, 2020). Therefore, a comprehensive treatment using different therapeutic strategies with immunotherapy may bring hope to pancreatic cancer patients (Schizas et al., 2020).

The patient stopped the medication in early October 2022 for financial reasons, and she was subsequently transferred to the hospital for the treatment due to tumor progression. From her tumor marker changes and abdominal CT results, it showed (Figures 4, 5) that the effect of Tegio chemotherapy was not satisfactory and the efficacy was much less than that of lenvatinib and icaritin soft capsules. Although the patient had to switch drugs for economic reasons, it provided clinical application evidence and reference for the subsequent combination treatment with targeted and immune-related drugs in advanced pancreatic cancer.

It is noteworthy that there were two sudden onsets of acute pancreatitis during the treatment in this case. Icaritin soft capsules as a new class I drug were launched in May 2022, the total number of users is currently small, and no reports related to triggering pancreatitis have been seen in phase III clinical studies, but cases of lenvatinib leading to pancreatitis have been reported (Kawakami et al., 2018). Therefore, it cannot be excluded that patient’s two acute pancreatitis were related to the application of lenvatinib. The safety of the combined application of the two drugs was positive throughout the treatment, with no serious complications. Moreover, because it was administered orally, the patient complied well and never discontinued the drug except for economic reasons, confirming the safety of the drug.

## Summary

Overall, the efficacy of icaritin soft capsules in combination with lenvatinib in this case of advanced pancreatic cancer is very remarkable. Also, because icaritin soft capsules have minimal effects on liver and kidney function and bone marrow suppression, it is well suited for combination with targeted or immune drugs for the treatment of advanced malignancies, bringing new hope to patients with advanced pancreatic cancer who are unable or refuse to receive first-line treatment with chemotherapy, at least, as an alternative treatment.

## Data Availability

The raw data supporting the conclusion of this article will be made available by the authors, without undue reservation.
